# Schools as smoke-free zones? Barriers and facilitators to the adoption of outdoor school ground smoking bans at secondary schools

**DOI:** 10.1186/s12971-016-0076-9

**Published:** 2016-03-29

**Authors:** A.D. Rozema, J.J.P. Mathijssen, M.W.J. Jansen, J.A.M. van Oers

**Affiliations:** Department Tranzo, Tilburg University, P.O. Box 90153, Tilburg, 5000 LE The Netherlands; Academic Collaborative Centre for Public Health Limburg, Public Health Service South Limburg (GGD ZL), Geleen, The Netherlands; Department of Health Services Research, School for Public Health and Primary Care CAPHRI, Maastricht University, Maastricht, The Netherlands; National Institute for Public Health and the Environment (RIVM), Bilthoven, The Netherlands

**Keywords:** Smoke-free, Adoption, Tobacco control policy, Health promotion, Secondary school

## Abstract

**Background:**

Whereas smoking bans inside secondary school buildings are relatively widespread, a smoking ban for the outdoor school grounds is less common. Therefore, this study investigates why many secondary schools fail to adopt an outdoor school ground smoking ban. The aim is to elucidate the perceived barriers and facilitators of stakeholders at schools *without* an outdoor school ground smoking ban.

**Methods:**

Qualitative data were obtained from 60 respondents of 15 secondary schools. Semi-structured interviews were held with various key stakeholders and a thematic approach was used for analysis of the transcripts.

**Results:**

The perceived barriers and facilitators of the stakeholders fell into four categories: 1) socio-political characteristics (legislation and social norm), 2) school characteristics (policy, decision process, enforcement, resources, workforce conditions, communication and collaboration), 3) individual characteristics (support, knowledge, and target group), and 4) smoking ban characteristics (environmental factors, guideline recommendations, outcome expectations, and evidence).

**Conclusions:**

These findings highlight the importance of legislation for outdoor smoking bans. Moreover, collaboration, communication and involving stakeholders during an early stage of the process should be stimulated, as this might increase adoption. These results can be applied in future studies on outdoor tobacco control policies; moreover, they may facilitate tobacco control initiatives leading to more smoke-free environments to further protect youth from the harmful effects of tobacco.

## Background

Tobacco use is a leading cause of morbidity and mortality worldwide. Despite the implementation of various tobacco control interventions (e.g. increased taxation, mass media campaigns, or smoke-free laws for indoor public places and workplaces) the prevalence of tobacco use remains problematic [1]. Given that the onset of smoking generally takes place during adolescence and results in increased tobacco involvement in adult life [2], tobacco control policies should focus on the prevention of smoking behavior of adolescents. A recent study showed that smoke-free environments have the potential to improve population health [3]. Moreover, smoke-free environments may not only reduce teenage smoking, but also exposure to second-hand smoke [4–6]. Therefore, implementing smoking bans seems promising to reduce and prevent tobacco use among adolescents.

Due to mandatory attendance, secondary schools are a potential setting for implementing smoking bans to prevent tobacco use among adolescents. Moreover, schools can play a key role in tobacco interventions as adolescence is a critical time for acquiring new patterns such as smoking initiation [7]. In fact, smoking rates among adolescents are a reason for concern. For example, in the Netherlands 31 % of the adolescents are experimenters, 16 % have smoked in the past 4 weeks and 9 % are daily smokers [8]. In addition, in one study, 45 % of the smoking adolescents stated that school is the place where their smoking behavior most often takes place [9]. Although studies stress the importance of protecting the developing brain from exposure to tobacco products during adolescence [10, 11], only a few countries (i.e., Belgium, Finland, Australia, New Zealand, five provinces in Canada, and two states in the USA) have banned smoking at secondary school outdoor areas as well as the indoor areas [12].

In the present study, the definition of outdoor school ground smoking bans is based on three guidelines: i) the ban applies to the whole site (i.e., everywhere), ii) the ban applies to everyone, including students, staff and visitors, and iii) the ban should be displayed, e.g. in the school regulations and/or by signs [13]. A lack of legislation for a smoking ban on schools grounds (e.g. in the Netherlands) might explain the low percentage of such smoking bans, as schools are not obliged to implement the ban. Currently, in the Netherlands about 52 % of the secondary schools lack adoption and implementation of the smoking ban [14]. Although the number of studies on adoption of more general prevention programs and tobacco prevention programs using curricula at secondary schools is growing [15–19], few have explored the adoption process of smoke-free policies at secondary schools. Thus, the question arises what stops secondary schools from adopting an outdoor school ground smoking ban.

Several models have been developed to improve our understanding of the innovation process, such as adopting and implementing an outdoor school ground smoking ban [20–23]. According to Fleuren et al. [21] the transition from the dissemination stage (i.e., people reading or hearing about the innovation) to the adoption stage (i.e., people acquiring and processing information and making decisions about the innovation) can be influenced by various determinants, divided into related categories [21, 24].

The present study explores which barriers and facilitators might affect adoption of an outdoor school ground smoking ban at secondary schools. This is important for two reasons. First, given that smoke-free environments internationally are increasingly important for improving general population health [3], identifying the barriers and facilitators can help in enacting smoke-free environments in school settings. In fact, a recent study emphasized the urgency for research on the adoption of tobacco control programs at schools, to more effectively facilitate tobacco prevention initiatives by policymakers and health professionals [25]. Second, to our knowledge, few studies have examined the adoption process of outdoor tobacco control policies in a school setting. The main aim of this study is to elucidate the adoption process of an outdoor school ground smoking ban by identifying the perceived barriers and facilitators of various stakeholders in secondary schools.

## Method

### Design

Qualitative methods are an effective way to explore the experiences and views of people with different roles in organizations [26]. Therefore, semi-structured interviews were held with key stakeholders in secondary schools to identify and elucidate the adoption process of an outdoor school ground smoking ban.

### Participants

The study took place in three Public Health Services regions in the northern, middle and southern part of the Netherlands, as these regions have a wide range of demographic characteristics (i.e., urbanization and ethnicity) and schools varying in their characteristics (i.e., education types and school size) (Table 1). In total, 31 secondary schools (with students aged 12–18 years) were contacted, 16 refused to participate and 15 participated. In most cases lack of time was the reason for non-participation. At these 15 schools, 60 stakeholders participated who differed in function, gender and smoking status (Table 2). Five types of stakeholders were interviewed, who are directly affected when implementing an outdoor school ground smoking ban: directors, non-teaching staff, teaching staff, parents and students.

**Table 1:** Characteristics of the participating schools

	No. of schools	
	*n* = 15	%
Size		
<500 students	4	27
500–1000 students	3	20
1000–1500 students	3	20
>1500 students	5	33
Urbanity		
Highly urbanized region	4	28
Urbanized region	6	40
Moderate urbanized region	3	20
Rural region	1	6
Highly rural region	1	6
Education type^a^		
Schools specialized in students with special needs^b^	1	6
Pre-vocational secondary education	9	60
Senior general secondary education	12	80
Pre-university education	12	80
Ethnicity		
West-European	12	80
Mixed	3	20

^a^Several schools had more than one education type

^b^School with students with psychiatric problems, physical, sensory or intellectual disabilities and behavioral disorders

**Table 2:** Characteristics of the stakeholders

	No. of stakeholders	
	*n* = 60	%
Function		
Directors	12	20
Non-teaching staff	15	25
Teaching staff	14	23
Parents	8	13
Students	11	18
Gender		
Male	38	63
Female	22	37
Smoking status		
Smokers	12	20
Ex-smokers	6	10
Non-smokers	42	70

### Procedure

The study was approved by the Psychological Ethics Committee of Tilburg University and informed consent was obtained from all participants included in the study. This study was conducted in collaboration with three of the 25 Public Health Services in the Netherlands in 2014. These services deliver screening and health promotion to meet the health needs of (amongst others) school populations. Three interviewers were employed by these Public Health Services and were trained and instructed by the first author (ADR) in data collection and interview techniques. The interviewers carried out the recruitment of the schools and the interviews with the stakeholders in their region. Secondary schools in the three regions were contacted by the interviewers and only schools *without* an outdoor school ground smoking ban were included. Moreover, variation in school size, urbanization and education type of the schools were taken into account during selection of the schools. Thereafter, in consultation with the school director, relevant stakeholders were selected within the school and asked to participate in the study, with a minimum of two and a maximum of five stakeholders at each school. This led to 60 interviewees in total. During selection of the stakeholders, their function and smoking status were taken into account.

Semi-structured interviews were conducted to explore the perceived barriers and facilitators of stakeholders with respect to a smoking ban in outdoor school grounds. Demographic variables were noted, i.e., function, age, smoking status, school size, percentage of smokers in school, education types, represented ethnicity at the school, and current smoking policy. Examples of questions during the interviews were: ‘What are the barriers to implement an outdoor school ground smoking ban?’ and ‘How easily could an outdoor school ground smoking ban be established?’. Data were recorded on a digital audio recorder and interviews were transcribed verbatim. The average duration of an interview was 34 (range 13–61) min.

### Analysis

Transcripts were analyzed using thematic analysis [27]. The coding was conducted by the first author. To establish the inter-rater reliability, all other authors coded 33 % of all transcripts separately and the codes were compared and discussed until consensus was reached. Thereafter, codes were revised and divided into general themes. Subsequently, any overlap between themes was reduced by creating overarching themes which conveyed the core essence of the themes. When the overarching themes were formed, discussions were held with all authors to verify the appropriateness and correctness of the themes. The framework of Fleuren et al. [21] was useful for this, as it represents the stages of innovation (e.g. dissemination and adoption) and the related categories of determinants. Therefore, the overarching themes were integrated in these four related categories of determinants. For the purpose of this study, the four categories (socio-political context, organization, the user and the innovation) formulated by Fleuren et al. [21] were further specified into socio-political, school, individual and smoking ban characteristics, and the overarching themes were divided into these four categories. An additional analysis was conducted to explore the different views held by the stakeholders (i.e., which stakeholders perceive which barriers or facilitators). Furthermore, member checks (i.e., sending stakeholders a summary of their interview and asking them to confirm that this accurately reflects their statements) were conducted with all stakeholders for interpretive validation [28]. Data were analyzed using the software package Atlas-Ti 7.

## Results

**Fig. 1:**
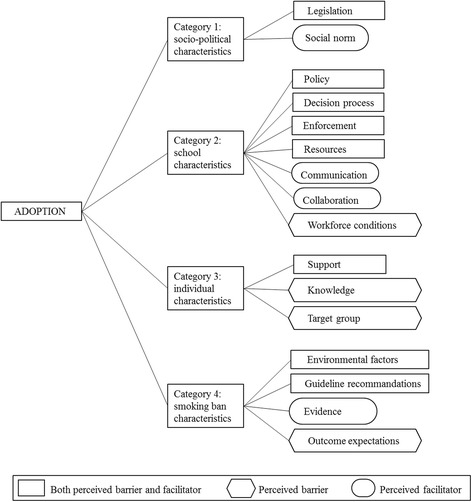
Perceived barriers and facilitators to the adoption of a smoking ban divided into four categories

A total of 16 overarching themes of perceived barriers and facilitators were identified for an outdoor school ground smoking ban, divided into four related categories, i.e., socio-political characteristics, school characteristics, individual characteristics and smoking ban characteristics (Figure 1). The results show that the perceived barriers and facilitators were often two sides of the same coin (e.g., lack of support as a barrier, and the need for support as a facilitator). However, workforce conditions, knowledge, target group and outcome expectations were only identified as perceived barriers, whereas social norm, communication, collaboration and evidence of the effectiveness of the smoking ban, were only identified as facilitators. The category ‘school characteristics’ included the highest number of perceived barriers and facilitators.

- Category 1: Socio-political characteristics
  - In the category socio-political characteristics, legislation was reported as a barrier (i.e., lack of legislation) and as a facilitator (i.e., need for legislation). For example, stakeholders reported that i) enacting legislation for outdoor school ground smoking bans at secondary schools, ii) stricter legislation for tobacco use of adolescents, and iii) government guidelines on outdoor school ground smoking bans, would facilitate adoption. Moreover, lacking this type of legislation and lacking legislation for public areas around secondary schools, hinders adoption. Furthermore, according to stakeholders, the social norm for outdoor smoking bans should be strengthened (e.g., by mass media) as this is seen as a facilitator: i.e. when outdoor smoking bans are accepted as normal and considered appropriate in society, this will enhance adoption.
- Category 2: School characteristics
  - In this category, 7 overarching themes of perceived barriers and facilitators were distinguished. As a first theme, policy is mentioned by stakeholders as both a barrier and a facilitator. Other priorities of the school (e.g., bullying, nutrition), a laissez-faire culture (i.e., low interference with the activities of students) and a policy which prohibits students leaving the school ground during school time (i.e., blocking the possibility to smoke causes problems among smokers), function as barriers. Conversely, a policy which prohibits leaving the school ground is also mentioned as a facilitator, as enforcement might then be easier. A tailored, stepwise and comprehensive implementation approach is seen as a facilitator. In addition, a well-chosen moment for implementation is also considered a facilitator, e.g., the start of a new school year.
  - Second, the decision process at secondary schools is considered as both a barrier and a facilitator. For example, a negative attitude of decision-makers towards an outdoor school ground smoking ban is seen as a barrier. Similarly, stakeholders did not expect a smoking ban to be adopted when the decision-makers themselves were smokers. Furthermore, not only a bottom-up decision approach but also a top-down decision approach is considered a facilitator. Nevertheless, stakeholders more frequently referred to a bottom-up approach than to a top-down approach.
  - Third, enforcement is mentioned as both a barrier and a facilitator. For example, stakeholders mentioned enforcement as a barrier because, due to additional pressures on staff etc., difficulties were expected with the enforcement of the ban.
    -
      *Only the actual enforcement prevents us from implementing the smoking ban, just the enforcement. That’s the only problem (*Director, smoker, #31*).*
  - Furthermore, some stakeholders mentioned that ratification of the director and strict enforcement by all staff members would facilitate the adoption of the smoking ban.
  - Fourth, the availability of resources is considered both a barrier and a facilitator. A lack of resources, e.g. finances (e.g., to make/place signs, etc.), staff and time, were reported as barriers to adopt the smoking ban.
    -
      *Implementing the smoking ban would take up far too much of our time. People forget that we’re a school, focusing on the education of students. This takes all our time and attention, together with all the other things that we have to do. So I, as a director, don’t want to invest any time at all on it.* (Director, non-smoker, #59)
  - On the other hand, sufficient finances and time were mentioned as facilitators. For example, receiving adequate funding for implementation would be a facilitator.
  - Fifth, the working conditions are considered a barrier; for example, there is insufficient staff to deal with an increase in workload due to a new task (e.g., enforcement of the ban). Furthermore, the employment terms of smoking personnel must be taken into account, e.g., personnel must have the opportunity to smoke during the breaks (i.e. their private time).
  - Sixth, communication is mentioned as a facilitator. For example, information and education provided by schools or external organizations are reported as facilitators. Stakeholders would like to start projects or workshops which increase awareness of the harmful effects of smoking and underpin implementing an outdoor school ground smoking ban.
    -
      *We’d like to receive information from the local Public Health Services. They should inform staff, parents and students about the legislation, the harmful effects of smoking and how to deal with it by implementing an outdoor school ground smoking ban. Then we’ll be able to continue moving forward to an outdoor school ground smoking ban.* (Teacher, non-smoker, #32)
  - Finally, collaboration is mentioned as a facilitator, not only within the school (e.g., collaboration within the school resulting in a cohesive team) but also with other schools (e.g., collaborating with other schools by sharing experiences/best practices) and with other external organizations (e.g., receiving counseling and implementation instructions).
- Category 3: Individual characteristics
  - In the category ‘individual characteristics’ three themes of perceived barriers and facilitators were identified. Firstly, support was both reported as a barrier (i.e., lack of support of smoking staff, smoking students and parents) and as a facilitator (i.e., need for support of smoking staff, smoking students, parents and residents). Without support for an outdoor school ground smoking ban, stakeholders do not foresee adoption.
    -
      *I’ve said it many times: I think it’ll be tough confronting the smoking staff - the smoking ban will provoke resistance from the smoking staff.* (Teacher, non-smoker, #19)
  - On the other hand, support will facilitate adoption. Secondly, lack of knowledge about the concept and about implementation are mentioned as barriers. Lastly, an ‘inappropriate’ target group is considered a barrier: e.g. if a school has students with severe problems, stakeholders expect aggression and rebelliousness when adopting an outdoor smoking ban.
- Category 4: Outdoor school ground smoking ban characteristics
  - Four themes were identified in the category of the smoking ban itself (i.e., what makes it difficult to adopt and what would simplify adoption). First, some environmental factors are impediments and some underpin adoption of an outdoor school ground smoking ban. For example, a large school ground and/or no clear demarcation of the school premises hinders enforcement and thus adoption. Moreover, an outdoor school ground smoking ban is irrelevant when a school does not have its own premises. Changing the school ground area (e.g., new construction, placing signs, removing ashtrays, etc.) and a vignette (i.e., an acknowledgment of a smoke-free school) are considered as facilitators in the category of environmental factors.
  - Second, according to stakeholders, guideline recommendations for an outdoor school ground smoking ban should be more flexible. For example, the guideline ‘Smoking is prohibited for everyone’ should not apply to staff or visitors who smoke. An exception must be made for adults, e.g., a place out of sight from the students but on the school grounds.
    -
      *When implementing the smoking ban, I think we should allow pupils aged 16 to 18 to smoke only with permission from their parents or only allow everyone who’s older than 18 years to smoke on the school grounds, because I think teachers shouldn’t be deprived of smoking.* (Pupil, smoker, #52)
  - Thirdly, a variety of negative outcome expectations are often reported by stakeholders as a barrier. For example, non-compliance of students and turbulence in the school (i.e., a smoking ban will cause conflicts). Moreover, stakeholders expect smokers to disappear from sight when leaving the school ground to smoke a cigarette, which may increase the risk of truancy and use of drugs, and/or may cause nuisance in the neighborhood. These negative outcome expectations may prevent adoption.
    -
      *If an outdoor school ground smoking ban is implemented, then students will smoke their cigarettes outside the school premises, which will increase littering in the neighborhood. That’s one of the biggest obstacles: the cigarette butts and students making a mess.* (Parent, smoker, #10)
  - Furthermore, a negative image of the school is mentioned as an outcome expectation: stakeholders expect smokers will smoke outside the school entrance when they are prohibited from smoking *on* the school premises; this may give a negative impression to people entering the school. Finally, evidence based on research demonstrating the effectiveness of the outdoor smoking ban on tobacco use of adolescents is considered a facilitator.

### Differences between stakeholders

All five types of stakeholders differed in their views regarding barriers and facilitators. Directors and parents valued outcome expectations as a barrier, while non-teaching and teaching staff and students also mentioned lack of support as a barrier. Directors and students valued collaboration as a facilitator; non-teaching and teaching staff reported communications as a facilitator; and parents mentioned legislation as a main facilitator. Smokers reported lack of support, workforce conditions and outcome expectations as barriers, and collaboration and communication as facilitators.

## Discussion

Until now, there is a lack of research on the adoption process of outdoor tobacco control policies in school settings, based on identifying the perceived barriers and facilitators [15–19, 25]. The present study provides insight into the perceived barriers and facilitators of stakeholders at secondary schools that affect the adoption of outdoor school ground smoking bans. Several barriers and facilitators were identified, divided into four categories: socio-political characteristics, school characteristics, individual characteristics, and characteristics of the smoking ban. Changes that positively affect adoption are needed, as a smoke-free environment in secondary schools seems promising to improve the general health of adolescents and may reduce teenage smoking and exposure to second-hand smoke [4–6].

Closer analysis of the different views of stakeholders reveals interesting insights. First, no substantial differences were found between the reported barriers and facilitators of non-teaching staff and teaching staff. Second, all main users of an outdoor school ground smoking ban (i.e., non-teaching staff, teaching staff and students) mentioned lack of support as a barrier. Third, negative outcome expectations were mentioned by directors (i.e. the stakeholders responsible for school policies). Fourth, smokers reported lack of support, workforce conditions and negative outcome expectations as barriers, since the smoking ban might threaten their own smoking behavior. Also, according to the smokers, communication and collaboration would facilitate adoption. Results show that the differences between the stakeholders should be taken into account when aiming to enhance adoption.

According to all stakeholders, there is a need for stricter legislation in the socio-political category. Enacting legislation depends on public acceptance of the outdoor smoking ban. For example, Diepeveen et al. [29] reported that public acceptance of government interventions is the highest for low intrusive interventions and when they target behavior of others, rather than the participants’ own behavior. In the context of an outdoor school ground smoking ban, the extent of public acceptance will probably be high because, in the present study, the stakeholders themselves (i.e., the targets) stated their preference for legislation. Moreover, Jaine et al. [30] reported that the support of adolescents for outdoor smoking bans increased from 51 % in 2009 to 59 % in 2011 and that, based on these results, the government should enact legislation. However, Widome et al. [31] report that public support alone is not enough for enacting legislation, but that multiple factors determine public health policy decisions. Nevertheless, in some countries (such as Belgium, Finland, Australia, and some states in Canada and the USA), legislation for an outdoor school ground smoking ban has already been implemented [12].

Results of the present study also suggest that collaboration and communication (not only information/education provided by the school or external organizations, but also mass media in the socio-political category) might increase adoption. Indeed, meetings with key stakeholders, educational presentations and media outreach is associated with tobacco policy change [32]. In line with our results, another study showed that shared decision-making (e.g., community participation and collaboration) enhances adoption of smoking bans [18]. These findings emphasize the importance of the involvement of several relevant stakeholders or parties at an early stage, resulting in community ownership which, in turn, establishes sustainability of an innovation [33]. In other words, in the context of secondary schools, collaboration, communication and involving stakeholders during the process might be essential to increase adoption of an outdoor school ground smoking ban.

Lack of knowledge on the individual category might influence the adoption process. However, a distinction must be made between lack of knowledge about the concept (i.e., knowledge about what an outdoor school ground smoking ban implies) and lack of knowledge about implementation, as knowledge about the concept is a precondition for adoption. That is, without knowledge about the concept, stakeholders of secondary schools will not acquire and process information and make decisions about the smoking ban. Apparently, in the present study some schools were not sufficiently informed about the concept and therefore not yet in the dissemination stage (i.e. people read/heard about a smoking ban), which is an essential stage before the adoption stage [21].

Furthermore, the results of this study show that several factors in the smoking ban category played a role in the adoption process. Flexibility of the guidelines (i.e., not everyone has to adhere/staff and visitors should be condoned) is needed to enhance the adoption rate, according to the stakeholders. Similar to our results, Durlak & DuPre [18] showed that adaptation plays an important role in innovations. It appears that users of an innovation often replicate some parts and modify other parts and that some degree of innovation adaptation is unavoidable [34]. However, adaptations can influence outcomes both positively and negatively [18]. For example, adaptation might improve non-smoking behavior among students, in other cases it might undermine the credibility of the ban if smoking staff is excused, since teachers are recognized as role models and this influences the smoking behavior of adolescents [35]. Future research should address the outcomes and effectiveness of an outdoor school ground smoking ban when there is some degree of adaptation of the smoking ban, since the literature showed mixed results [36, 37]. Additionally, the level of support of smoking staff should be measured both when restricting teachers smoking and when condoning them, as even after implementation of a smoking ban the level of support often remains low among smoking staff [38], and condoning them might increase support.

Some limitations should be considered when interpreting the results. First, differences between the various schools were not revealed with respect to the perceived barriers and facilitators (e.g., schools differing in size, education level). However, because an earlier study showed that adoption of a tobacco-free policy did not differ substantially between different types of schools [32], the differences between schools are probably small. A second limitation is that only Dutch secondary schools with Dutch representatives were included. Future research should address non-Dutch schools with reference to cultural diversity and international comparison. Although these limitations are relevant, we did include a large and diverse group of schools and stakeholders which led to maximal exploration of the barriers and facilitators. Overall, the wide variation and the richness of the data contribute to the generalizability of our findings [39] and offer new insight into the processes of adoption of an outdoor smoking ban in a school setting.

## Conclusion

The findings highlight the importance of legislation for outdoor smoking bans in a school setting. Policymakers are advised to develop and implement legislation, as this may help normalize outdoor smoking bans in school settings. Furthermore, school-wide promotions, media messages and meetings with key stakeholders are important components for adoption. Collaboration, communication and involving stakeholders at an early stage of the process should be stimulated, as this might be essential to increase adoption. In general, the findings of this study can be used in future subsequent adoption studies of outdoor tobacco control policies. However, most importantly, the findings of this study can facilitate tobacco control initiatives which, in turn, might result in more smoke-free environments and additional protection of youth from the harmful effects of tobacco.

## Ethical approval

All procedures performed in this study were in accordance with the ethical standards of the institutional research committee and with the 1964 Helsinki declaration and its later amendments or comparable ethical standards. Informed consent was obtained from all participants, including consent to publish.
